# Comparison of Two Process Schemes Combining Hydrothermal Treatment and Acidogenic Fermentation of Source-Separated Organics

**DOI:** 10.3390/molecules24081466

**Published:** 2019-04-13

**Authors:** Long Lin, Ehssan Hosseini Koupaie, Armineh Azizi, Amir Abbas Bazyar Lakeh, Bipro R. Dhar, Hisham Hafez, Elsayed Elbeshbishy

**Affiliations:** 1 Department of Civil and Environmental Engineering, University of Alberta, 116 Street NW, Edmonton, AB T6G 1H9, Canada; llin2@ualberta.ca (L.L.); bipro@ualberta.ca (B.R.D.); 2 Environmental Research Group for Resource Recovery, Department of Civil Engineering, Faculty of Engineering, Architecture and Science, Ryerson University, 350 Victoria Street, Toronto, ON M5B 2K3, Canada; ehssan.hosseini.k@gmail.com (E.H.K.); armineh.azizi@ryerson.ca (A.A.); amir.bazyar@ryerson.ca (A.A.B.L.); Hisham.Hafez@greenfield.com (H.H.); 3 Greenfield Global, 275 Bloomfield Road, Chatham, ON N7M 0N6, Canada

**Keywords:** acidogenic fermentation, hydrothermal treatment, source separated organics, volatile fatty acids, particulate organics solubilization, microbial community analysis

## Abstract

This study compares the effects of pre- and post-hydrothermal treatment of source- separated organics (SSO) on solubilization of particulate organics and acidogenic fermentation for volatile fatty acids (VFAs) production. The overall COD solubilization and solids removal efficiencies from both schemes were comparable. However, the pre-hydrolysis of SSO followed by acidogenic fermentation resulted in a relatively higher VFA yield of 433 mg/g VSS, which was 18% higher than that of a process scheme with a post-hydrolysis of dewatered solids from the fermentation process. Regarding the composition of VFA, the dominance of acetate and butyrate was comparable in both process schemes, while propionate concentration considerably increased in the process with pre-hydrolysis of SSO. The microbial community results showed that the relative abundance of *Firmicutes* increased substantially in the fermentation of pretreated SSO, indicating that there might be different metabolic pathways for production of VFAs in fermentation process operated with pre-treated SSO. The possible reason might be that the abundance of soluble organic matters due to pre-hydrolysis might stimulate the growth of more kinetically efficient fermentative bacteria as indicated by the increase in *Firmicutes* percentage.

## 1. Introduction

In the world, about 1.2 billion tonnes of food are wasted every year through the food supply chain, accounting for one-third of the food production [1]. In Canada, more than 27 million tonnes of food waste are disposed of annually [2]. If not adequately managed, the large amount of organic waste may cause significant social, environmental, and economic challenges. Source separated collection has been an effective management strategy to separate organic waste from other waste streams at the source, thereby minimizing the contamination of organic waste for the downstream process. Source-separated organics (SSO) is the term used to describe the organic waste stream collected at the source [3]. Besides food waste, SSO also contains other organic components, including yard trimmings (grass, leaves, etc.), fibres (paper towels, napkins, tea bags, etc.) and wood waste [4].

The bioconversion of SSO to various value-added products (e.g., CH_4_, H_2_, volatile fatty acids), and thereby reduce waste for ultimate disposal has attracted increasing attention. Specifically, waste-derived volatile fatty acids (VFA) production has been emerging due to the diverse application of VFAs [5]. The biological steps involved in VFA production are hydrolysis and acidogenesis. Hydrolysis, in which bacteria use enzymes to break down particulate matters or macromolecule into soluble compounds or monomers, is usually considered as the rate-limiting step. Pre-treatment of organic waste can be used to enhance the hydrolysis of particulate matters [6]. Among various pre-treatment methods, hydrothermal pre-treatment is considered environmentally friendly because no chemical addition is needed. The hydrothermal pre-treatment uses high temperature and pressure to increase the ionized products of water, which facilitate the hydrolysis of particulate matters into the soluble phase [7]. Also, the associated thermal energy can potentially be recovered at the end of the process, thereby reducing energy demand [8]. Moreover, various types of waste heat from boiler steam or flue gas can be utilized for the hydrothermal process at no additional cost [9].

Previous studies have reported positive impacts of hydrothermal pre-treatment of food waste on fermentative VFA production. For instance, a study showed hydrothermal pre-treatment of food waste at 160 °C for 30 min, and achieved 43% increase in SCOD and 55% increase in VFA after fermentation for 15 days [10]. Yin et al. [10] also reported that hydrothermal temperature higher than 170 °C led to the formation of some toxic and non-biodegradable products despite higher COD dissolution. Similarly, another study also examined the effect of hydrothermal pre-treatment of kitchen waste and obtained 1.3 times higher VFA production after fermentation for 21 days [11]. The compositions of SSO vary considerably from food waste, which could profoundly affect the hydrothermal pre-treatment and subsequent fermentation performance. However, most of these studies focused on food waste and did not identify detailed information on microbial composition during fermentation of pre-treated substrates. The differences between microbial communities involved in fermentation of SSO and pretreated SSO remained to be determined. Furthermore, as the hydrothermal process mainly enhances the solubilization of particulates, it may instead be used as post-treatment after fermentation to target only the solid fraction of fermented effluent, which has rarely been investigated. Thus, it is still unknown whether the order of applying the hydrothermal process affects solubilization of particulate organics and VFA production when combining with acidogenic fermentation.

Therefore, the objective of this study was to compare the performance of the hydrothermal process used as pre-treatment and post-treatment on solubilization of particulate organics as well as VFA production from SSO. The main parameters evaluated were solubilization of particulate matters, solids reduction, and VFAs production and composition. The microbial communities were also examined in the fermented effluent with/without hydrothermal pre-treatment to elucidate the possible VFA production pathways. Lastly, the implications of this study were also discussed comparing to literature. This study first compared the two process schemes combining hydrothermal treatment and acidogenic fermentation of SSO and provided new information into microbiome that drives fermentation with pretreated SSO. The findings may help advance understanding of fermentation process and operation integrating with hydrothermal treatment.

## 2. Results and Discussion

### 2.1. Solubilization of Particulate Matters

**Figure 1:**
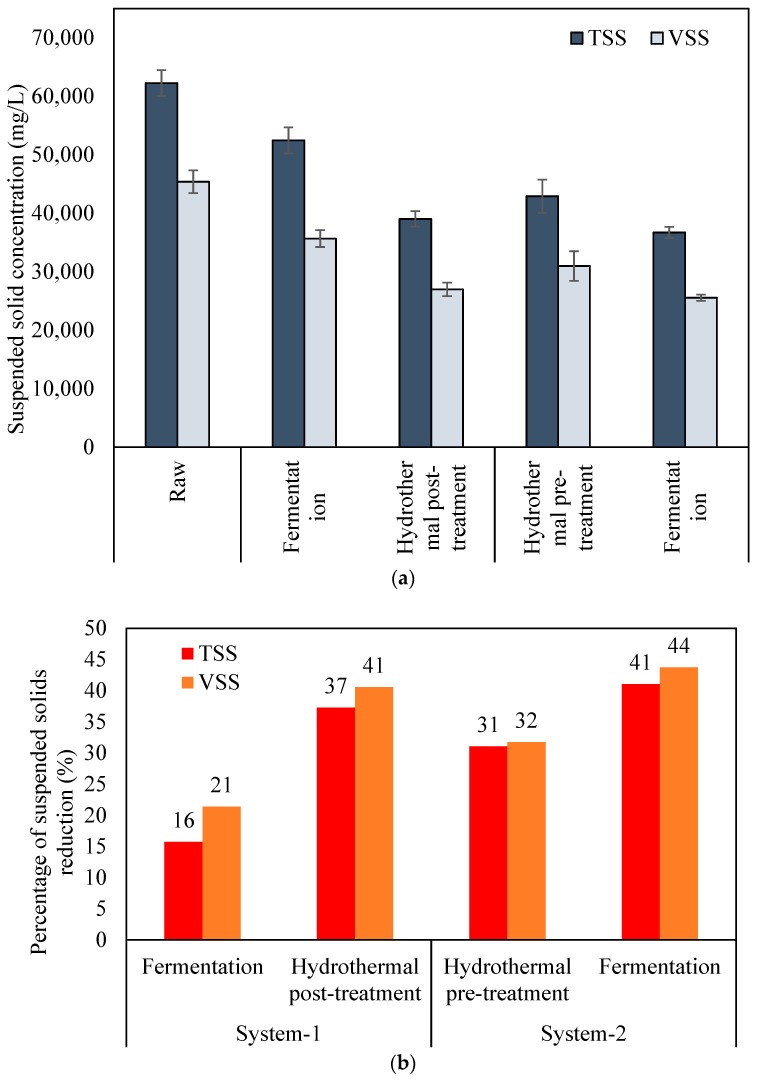
(**a**) The concentrations of suspended solids and (**b**) suspended solid reduction efficiency in raw, treated, and fermented SSO. (TSS: Total Suspended Solids, VSS: Volatile Suspended Solids)

Figure 1 shows the concentrations and reduction efficiencies of suspended solids in raw, treated, and fermented SSO samples. The initial TSS and VSS concentrations in raw SSO were 62,000 mg/L and 45,400 mg/L, respectively. In system-1, raw SSO was fermented followed by post-hydrolysis of dewatered solids from the fermentation process. Conversely, in system-2, raw SSO was subjected to hydrothermal pre-treatment followed by fermentation. Both systems showed a step-wise decrease in TSS and VSS concentrations after each step of treatment. Moreover, each step in system-2 showed a superior performance in suspended solids reduction than the corresponding step in system-1. After fermentation (system-1), the TSS and VSS concentrations decreased to 52,500 mg/L and 35,700 mg/L respectively. The subsequent hydrothermal post-treatment further decreased the TSS and VSS concentrations to 39,000 mg/L and 27,000 mg/L, respectively. In system-2, the TSS and VSS concentrations first substantially reduced to 43,000 mg/L and 31,000 mg/L after thermal pre-hydrolysis; then, further reduced to 36,700 mg/L and 25,500 mg/L after fermentation, respectively. These results indicated that both pre- and post- thermal hydrolysis processes efficiently hydrolyzed particulate organics. The thermal hydrolysis process alone (31–32%) had a superior effect on the reduction of suspended solids of SSO than fermentation alone (16–21%). Notably, a major portion of the solid reduction in system-2 was contributed by the pre-hydrolysis step. Nonetheless, overall solids removal efficiencies from both schemes were comparable despite the slight advantage showed in system-2 (41–44% vs. 37–41%).

**Figure 2:**
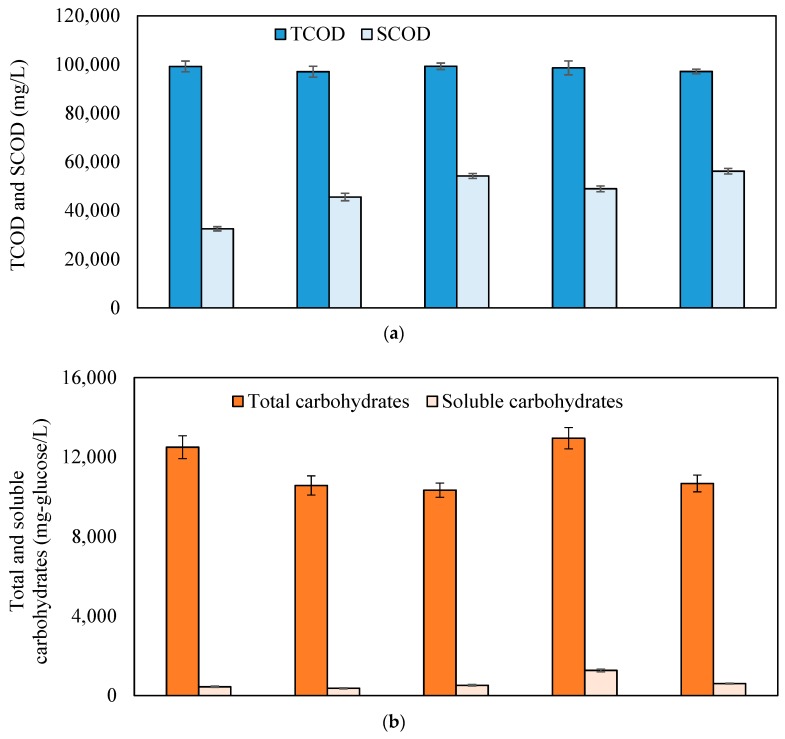

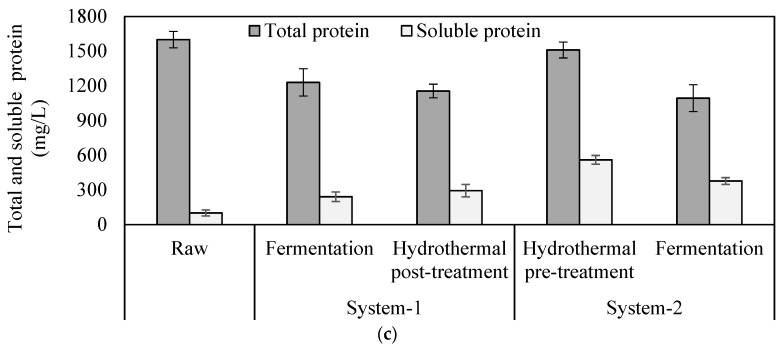
Concentrations of (**a**) TCOD and SCOD, (**b**) total and soluble carbohydrates, and (**c**) total and soluble protein in raw, treated, and fermented SSO. (TCOD: Total Chemical Oxygen Demand, SCOD: Soluble Chemical Oxygen Demand, SSO: Source Separated Organics)

Figure 2 shows concentrations of TCOD and SCOD, total and soluble carbohydrate, and total and soluble protein. The degrees of COD, carbohydrate, and protein solubilization are provided in the Supplementary Information (Figure S1). The initial concentrations of SCOD (32,500 mg/L), soluble carbohydrate (442 mg/L), and soluble protein (100 mg/L) in the raw SSO accounted for 32.8%, 3.5%, 6.3% of the TCOD, total carbohydrate, and total protein, respectively. Similar to solids reduction, both systems showed a step-wise increase in SCOD after each step of treatment, and each step in system-2 showed a better performance in terms of COD solubilization than the corresponding step in system-1. The fermentation in system-1 first increased the SCOD concentration from 32,500 mg/L to 45,600 mg/L, resulting in a 20% degree of solubilization. The subsequent post-hydrolysis further increased the SCOD to 54,200 mg/L, leading to an overall degree of solubilization of 33%. In comparison, the pre-hydrolysis in system-2 first increased the SCOD concentration to 49,000 mg/L, resulting in a 25% degree of solubilization. The subsequent fermentation process further increased the SCOD to 56,200 mg/L, corresponding to an overall degree of solubilization of 35%. Thus, the COD solubilization results were consistent with the trend observed for solids reduction. Furthermore, it is worth noting that the TCOD remained almost constant in both systems, indicating that negligible COD (<2%) were released as gaseous by-products (e.g., H_2_, CO_2_) from the fermentation or hydrolysis process, which was in line with the observation of negligible H_2_ production in this study. As shown in Figure 2b,c, fermentation reduced the concentration of soluble carbohydrate and soluble protein in both process schemes, mainly due to the fermentation of these compounds to lower molecular organics, including various volatile organic acids.

The post-hydrolysis of dewatered solids from the fermentation process slightly increased the soluble carbohydrate and protein in system-1, while the pre-hydrolysis of SSO in system-2 considerably increased the concentrations of soluble carbohydrate and protein. According to the literature, the high temperature in the hydrothermal pre-treatment process hydrolyzed macromolecules (e.g., starch, cellulose, and hemicellulose, protein) into small molecular matter like oligosaccharide and monosaccharide (e.g., glucose and xylose), leading to the increase of soluble carbohydrates and protein [5,9]. Meanwhile, some soluble sugars and amino acids were further degraded into short-chain VFAs, such as acetic acid, thus decreasing the total carbohydrates/proteins. Lipids have been reported to be relatively stable under the hydrothermal process and fermentation [12]. Organic matters present in SSO are mostly carbohydrates, proteins, and lipids [13]. Therefore, the increase in the SCOD concentration of SSO during hydrothermal pre-treatment was most likely due to the solubilization of macromolecular organic compounds like carbohydrates and proteins. After fermentation, SCOD was increased while soluble carbohydrate and protein concentrations were decreased, which suggest that other intermediates (e.g., VFA) rather than soluble carbohydrate or protein were the main contributors of SCOD after fermentation.

### 2.2. Yields and Distribution of VFAs

**Figure 3:**
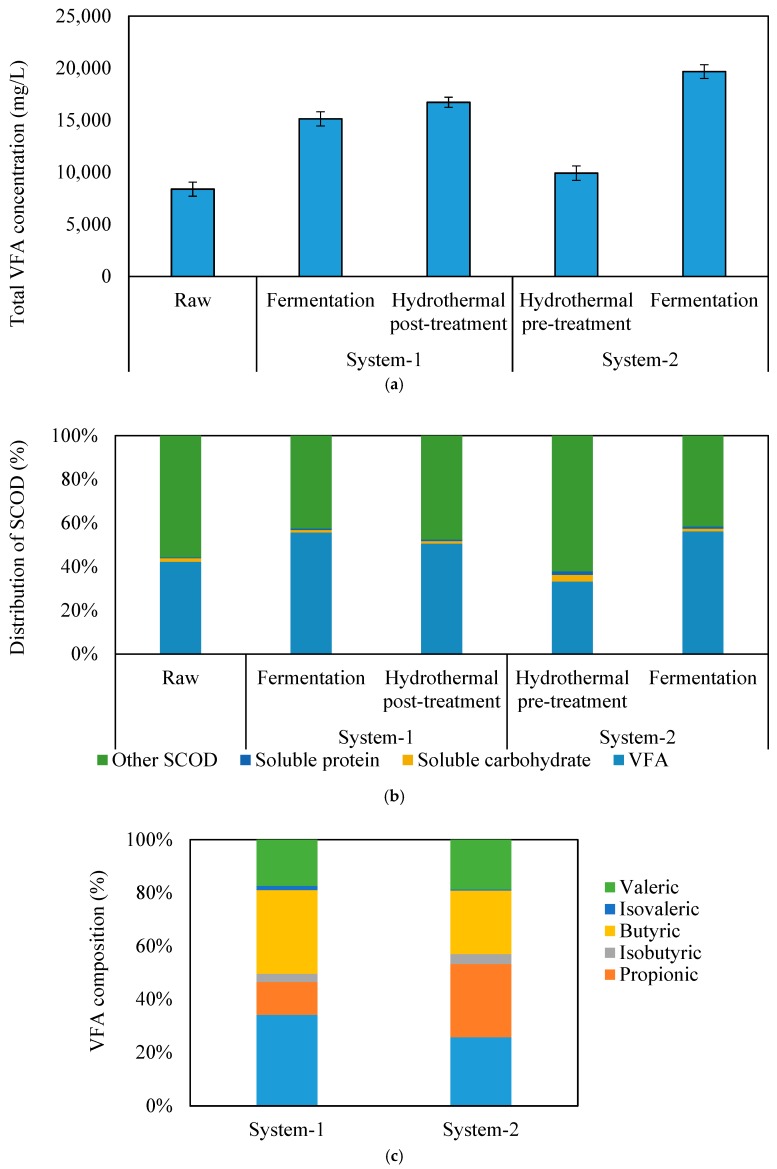
(**a**) The concentration of VFA, (**b**) distribution of SCOD component in raw, treated, and fermented SSO samples, and (**c**) composition of VFA in fermented SSO samples. (SCOD: Soluble Chemical Oxygen Demand, VFA: Volatile Fatty Acids, SSO: Source Separated Organics)

Figure 3a shows the total VFA concentrations in raw, hydrolyzed, and fermented SSO samples. The corresponding VFA yields and SCOD yields (mg/g VSS) are provided in the Supplementary Information (Table S1). The initial VFA in raw SSO was 8400 mg/L. In system-1, the VFA was significantly increased to 15,000 mg/L after fermentation, which was 80% higher compared to raw SSO. The subsequent hydrothermal process only further increased the VFA concentration slightly to 16,700 mg/L. In contrast, pre-hydrolysis in system-2 only slightly increased the VFA concentration (18%) in SSO. The subsequent fermentation substantially increased the VFA concentration to 19,700 mg/L with an overall increase of 135% compared to the raw sample. Nonetheless, the VFA production primarily credited to the fermentation process in both systems. Accordingly, the highest VFA yields (433 mg/g VSS) and SCOD yields (1240 mg/g VSS) were obtained from fermentation of pre-treated SSO in system-2, which were 135% and 73% higher than those in raw SSO samples, respectively (Supplementary Information, Table S1). This result indicated that the order of applying the hydrothermal process affected the VFA production. While combining hydrothermal process and fermentation, applying hydrothermal process as pre-treatment showed a slight advantage over as post-treatment, resulting in 18% higher VFA production. Because the hydrothermal process solubilized particulate to soluble phase, providing soluble organics for fermentation, thereby enhancing the VFA production during the fermentation process. Similar to VFA production, ammonia concentration (Supplementary Information, Figure S2) was substantially increased after fermentation, indicating the effective degradation of organic nitrogen (protein) during fermentation.

The highest VFA yield (433 mg/g VSS) attained in this study was further compared to those obtained in other studies. As shown in Table 1, VFA yields varied with operating conditions, such as hydrothermal temperature and holding time, and fermentation temperature and retention time. The substrate sources also had large impacts on VFA yields. Nonetheless, the VFA yield in this study is comparable to that reported by Ding et al., [12], but higher than that achieved by Li and Jin [14] and lower than those obtained by Yin et al., [10] and Yu et al., [11]. However, the VFA yield of SSO could be further improved by optimising other factors, such as HRT of fermentation, which should be investigated in future research.

Figure 3b shows the distribution of SCOD component in the raw, hydrolyzed, and fermented SSO samples based on the organic compounds, including VFA, soluble carbohydrate, and soluble protein. Among the soluble end products, VFA was the major contributor (56%) of SCOD after fermentation in both process schemes. In contrast, other SCOD rather than VFA was the main contributor (67%) of SCOD after pre-hydrolysis in system-2. In addition to soluble carbohydrates and soluble protein, other soluble SCOD may include alcohols, other organic acids, poly-phenols, furfural, etc. [9,15].

Figure 3c shows the composition of VFA in the fermented SSO samples from system-1 and system-2. In system-1, acetate (35%) and butyrate (30%) were the main products with a small production of propionate (12%). In system-2, besides the dominance of acetate (26%) and butyrate (22%), propionate (30%) was substantially increased compared to that in system-1. The difference in VFA compositions indicated that there might be different metabolic pathways for VFA production after pre-hydrolysis in system-2. According to the literature, acetate and butyrate were mainly produced from glucose fermentation [16]. Each glucose unit could be converted into either two acetate units and four hydrogen units via the acetate pathway or into one butyrate unit and two hydrogen via the butyrate pathway [17]. In contrast, propionate was mainly produced from the fermentation of amino acids [18] or various H_2_-consumption pathways [19]. Thus, the higher propionate fraction in system-2 might be produced by amino acid fermentation bacteria or by H_2_-consumption propionate producing bacteria. The dominance of propionate in VFAs has also been previously reported after the fermentation of thermally pretreated kitchen waste [14]. Meanwhile, previous studies have also shown that VFA composition in fermentation was highly dependent on the type of waste [12]. SSO characteristics markedly varied given the differences in origin, dietary customs, and collecting sites and seasons [20]. Therefore, the composition of VFAs in this study could be influenced by SSO characteristics.

### 2.3. Microbial Community

**Figure 4:**
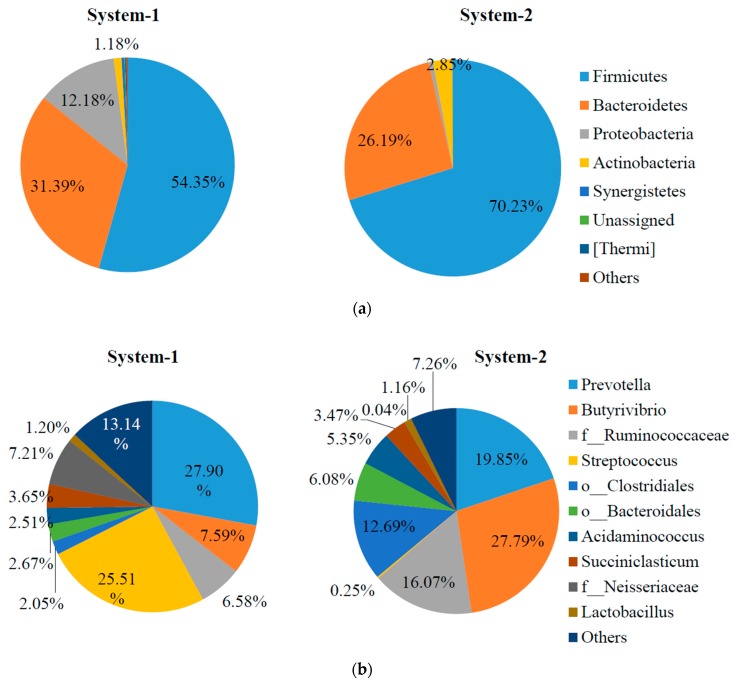
Relative abundance of bacterial composition at (**a**) phylum level and (**b**) genus level in fermentation process in system-1 and system-2. Note: those genera accounted for <1% of the total bacterial population were grouped into “others”. Those unidentified genera were represented by the lowest taxonomic level assigned, such as family (f_) or order (o_) level.

Bacterial community at the phylum level and genus level is shown in Figure 4. At the phylum level, *Firmicutes* was the most dominant one in both samples, but was considerably higher in relative abundance in the sample from system-2 (54% vs. 70%) (Figure 4a). *Firmicutes* contains various fermentative bacteria. *Bacteroidetes* was the second most dominant one in both samples (~30%). In system-2, *Actinobacteria* was also slightly higher in relative abundance (1% vs. 3%), while the relative abundance of *Proteobacteria* dramatically decreased (12% vs. 1%). 

At the genus level, *Prevotella* (*Bacteriodetes*) was dominant in both systems, and was relatively higher in relative abundance in system-1 (28% vs. 20%) (Figure 4b). Also, the percentage of *Streptococcus* (*Firmicutes*) was remarkably higher in system-1 (26% vs. 0.3%), which is well-known for lactic acid fermentation [22]. In contrast, *Butyrivibrio* (8% vs. 28%) and *Acidaminococcus* (2.5% vs. 5.4%) were higher in relative abundance in system-2. *Butyrivibrio* spp. contain butyric-acid-producing bacteria [23] and *Acidaminococcus* spp. contain amino-acids-degrading bacteria [24], both of which belong to the phylum *Firmicutes*. Previous studies have also reported the fermentation of model hemicelluloses by *Prevotella* strains and *Butyrivibrio fibrisolvens* [23]. Additionally, those unidentified genera sorted to the family *Ruminococcaceae* and the order *Clostridiales* (*Firmicutes*) were substantially higher in relative abundance in system-2, which contain a variety of fermentative bacteria [2,25]. These results indicated that there might be different metabolic pathways for VFA production in the fermentation process with pretreated SSO. One possible reason might be that the hydrothermal pre-treatment enhanced the soluble organic matters, thereby inducing the growth of various fermentative bacteria as indicated by the increased relative abundance in *Firmicutes*. Interestingly, the genus *Clostridium*, known as the main H_2_-producer in the fermentation process [22], was not detected with high relative abundance in either system, which was in line with the observation of negligible H_2_ production in this study.

## 3. Materials and Methods

### 3.1. SSO and Inoculum

In this study, the SSO was provided by the Disco Road Organics Processing Facility (Toronto, ON, Canada). This facility process SSO that consists of food waste, pet waste, houseplants, paper food packaging, diapers, and biodegradable plastics. At Disco facility, the raw materials first undergo visual inspection to remove the large items from the waste stream. After visual inspection, the waste streams enter a BTA^®^ hydro-mechanical system (BTA International GmbH, Pfaffenhofen, Germany) that transforms the non-homogeneous mixture of the waste into a semi-homogeneous pulp. The BTA process starts with a screening followed by hydropulpers to remove the unwanted materials (e.g., plastic bags, sand, metals, and glass shards) from the produced pulp. It is worth noting that the SSO utilized in this study was collected from the pulp stream after the BTA process. The fermentation inoculum was collected from a full-scale mesophilic anaerobic digester located in Ashbridge Bay wastewater treatment plant (Toronto, ON, Canada). The digester was operated at a temperature of 35–37 °C and fed with a mixture of primary and secondary municipal sludge. Table 2 summarizes the characteristics of the SSO and inoculum used in this study.

### 3.2. Experimental Design and Set-Up

The hydrothermal treatment of SSO was carried out using a high temperature (up to 330 °C), and high pressure (up to 1900 psi) stirred reactor with a maximum sample volume of 2 L (Parr 4848, Parr Instrument Company, Moline, IL, USA). The Parr hydrothermal reactor was equipped with an automated controller with auto-tuning capabilities that allows for accurate monitoring of both the heating and cooling parameters including target temperature, holding time (soak) as well as the heating/cooling rate. The reactor content (SSO) was continuously mixed with the aid of a mechanical mixer connected to a speed controller. The hydrothermal treatment parameters such as temperature and pressure were monitored on a real-time basis via the SpecView software version 3.1.144 (SpecView Corporation, Gig Harbor, WA, USA). In this study, for each round of treatment, 2 L of SSO was delivered into the reactor vessel. After sealing the vessel, the mechanical mixer was set to the rotational speed of 150 rpm. The mixer was kept running until the end of the cooling cycle. The hydrothermal temperature of 170 °C with a holding time of 30 min was used in this study, which was the favorable condition obtained from the previous study [13]. For this purpose, the temperature of SSO increased gradually at a rate of 3 °C/min until it reached the target temperature of 170 °C (Cycle 1). Next, the temperature was kept constant at 170 °C for 30 min (Cycle 2). Afterwards, the cooling cycle was started and continued until the temperature reached below 50 °C (Cycle 3). The hydrothermal treatment condition (temperature, pressure, and retention time) was selected considering the optimum hydrothermal treatment condition obtained among 15 different tested scenarios in an earlier study performed by the same research group [13].

**Figure 5:**
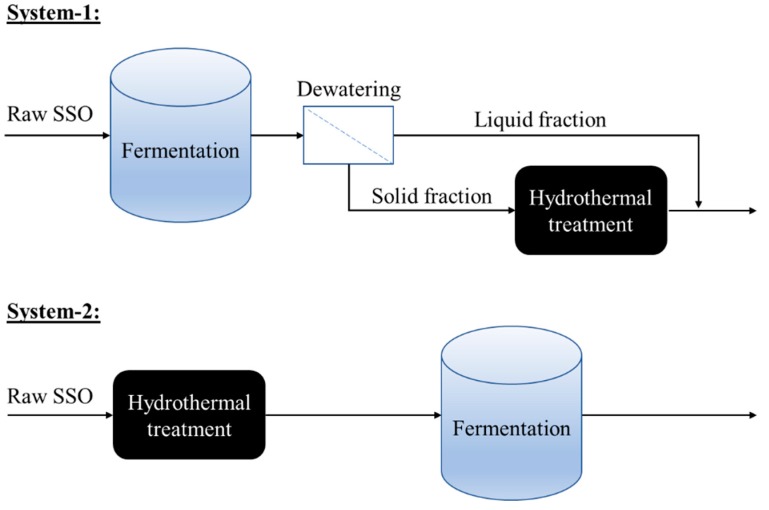
Schematic diagram showing the experimental set-up.

The experimental scheme of this study is shown in Figure 5. In system-1, the raw SSO was first fermented in a semi-continuous process. The fermenter effluent was then centrifuged for 30 min at 5000 rpm using a centrifuge (Sorvall Legend XT centrifuge, Fisher Scientific, Hampton, NH, USA) to separate the liquid and solid fraction of the fermented effluent. Then, the solid fraction of the fermented effluent was subjected to hydrothermal post-treatment. Afterwards, the liquid fraction (that was already separated through centrifugation) was mixed with the hydrothermally post-treated solid fraction to produce the final sample for analysis. In system-2, the raw SSO was first subjected to hydrothermal pre-treatment. Then, the pretreated SSO was used as the feedstock for a semi-continuous fermentation to produce a final sample for analysis.

Feeding of the fermenters was done semi-continuously (once every 24 h). Before starting the fermentation process, the content of the fermenters was purged for a duration of 5 min with nitrogen gas to achieve a completely anaerobic condition. The pH of the fermenters was kept in the range of 5.4–5.6 by controlling the pH of the feed (raw and pre-treated SSO) throughout the operation period using sodium hydroxide (NaOH) and hydrochloric acid (HCl). The fermentation process was conducted in a water bath set at a temperature of 38 ± 1 °C. The fermenter content was continuously mixed throughout the operation period at a rate of 150 rpm using a sealed rotating plastic shaft. Every 24 h, 500 mL of the fermentation effluent was taken out, and the fermenters were fed again with 500 mL of either raw or thermally pretreated SSO, resulting in hydraulic retention time (HRT) of 3 d. The feeding and decanting of the fermenters were done manually using a 500 mL aluminum syringe. Both the raw and thermally pretreated fermenters were operated for an initial period of 20 days to reach a steady-state condition and then the operation was continued for an additional 30 days. Eight samples (twice a week) were taken for analyses during the steady-state period.

### 3.3. Analytical Methods

The measurement of the samples solid content in terms of total solids (TS), volatile solids (VS), total suspended solids (TSS), and volatile suspended solids (VSS) was performed according to the Standard Methods [26]. The analysis of chemical oxygen demand (COD) was done through the closed reflux colorimetric method outlined by the Standard Methods. The carbohydrates and protein determination was conducted according to the colorimetric methods developed by Dubios et al. [27] and Frolund et al. [28]. The absorbance measurement of the samples was done using a spectrophotometer (DR 3900, HACH, Loveland, CO, USA) with the wavelengths of 600, 595, 490, 560, 650, 394, and 542 nm for the analysis of COD, protein, carbohydrates, ammonia, alkalinity, total nitrogen, and total phosphate, respectively. The COD, protein, and carbohydrate analyses were performed on both the total and soluble phases of the samples. To collect the soluble fraction for analysis, the samples were first centrifuged at 9000 rpm for 30 min using a Sorvall Legend XT centrifuge. Next, the liquid fraction (supernatant) of the centrifuged samples were filtered through 0.45 µm microfiber filters. The concentration of the total VFAs was determined calorimetrically using the TNT 872 kit (HACH). The VFAs composition was determined using a gas chromatograph (Varian 8500, Varian Inc., Toronto, ON, Canada) equipped with a fused silica column (30 m × 0.32 mm) and a flame ionization detector. Helium was used at a constant flow rate of 5 mL/min as the carrier gas. The temperatures of the detector and column were set at 250 and 110 °C, respectively.

### 3.4. Microbial Community Analysis

Microbial communities of the fermented effluent from both schemes were analyzed with high throughput 16S rDNA sequencing. The genomic DNA of the biomass samples were extracted using the PowerSoil^®^ DNA Isolation Kit (MoBio Laboratories, Carlsbad, CA, USA). The extracted DNA concentrations were quantified using a spectrophotometer (NanoDrop 2000C, Thermo Fisher Scientific, Waltham, MA, USA). The extracted DNA samples were stored immediately at −70 °C prior to performing Illumina Miseq sequencing at the Research and Testing Laboratory (Lubbock, TX, USA) using the bacterial primer set 357Wf: CCTACGGGNGGCWGCAG and 785R: GACTACHVGGGTATCTAATCC to target the 16S rDNA. The demultiplexed sequencing data were processed and analyzed using the Quantitative Insights Into Microbial Ecology (QIIME v2) software [29]. The sequences were denoised (dada2 method) to remove and/or correct noisy reads, remove chimeric sequences and singletons, and join denoised paired-end reads [30]. The denoised sequences were assigned to species-equivalent operational taxonomic units (OTUs) at a 97% sequence similarity level using the open-reference OTU picking method (vsearch method against 2013-08 Greengenes database) [31].

### 3.5. Calculations

The degree of solubilization of carbohydrate, protein, and COD were calculated to assess the hydrolysis efficiency (Equation (1)):

Degree of solubilization = (S_t_ − S_0_)/(T_0_ − S_0_) × 100% (1)

where, S_t_ is the concentration of soluble organic materials (carbohydrate, protein, or COD) in the treated SSO (mg/L), S_0_ is the initial concentration of soluble organic materials in the raw SSO (mg/L), and T_0_ is the initial concentration of total organic materials in the raw SSO (mg/L).

The yields of SCOD and VFA were calculated (Equation (2)):

Yield = S_t_/VSS_0_, expressed as mg/g − VSS (2)

where, S_t_ is the final concentration of SCOD or VFA in the treated SSO (mg/L), and VSS_0_ is the initial concentration of volatile suspended solids in the raw SSO (mg/L).

The conversion factors used for the calculation of COD of the carbohydrate (1.19 gCOD/g), protein (1.42 gCOD/g), acetate (1.07 gCOD/g), propionate (1.51 gCOD/g), butyrate (1.82 gCOD/g), and valerate (2.04 gCOD/g) were determined as previously described in the literature [32].

### 3.6. Statistical Analysis

The statistically significant effects of the independent variables (i.e., pretreatment temperature, pressure, and retention time) were tested by multi-factor analysis of variance (ANOVA) at a 95% confidence level (α = 0.05) using Minitab Software 17 (Minitab, LLC, State College, PA, USA). The ANOVA analysis was conducted by considering 2-level interaction effects of the independent variables. The Fisher’s least significant difference analysis was used to compare all pairs of means.

## 4. Conclusions

The process schemes of pre- and post-hydrothermal treatment of SSO were successfully performed and compared for solids removal and VFA yields. Results showed that solubilization of organic matters through pre-hydrolysis could boost subsequent acidogenic fermentation, resulting in an 18% increase in overall VFA yield over post-hydrolysis of fermented SSO. Both acetate and butyrate were dominant in both process schemes, while propionate was higher in the process with pre-hydrolysis. Meanwhile, various fermentative bacteria belonging to *Firmicutes* were higher in relative abundance in the process with pre-hydrolysis of SSO, suggesting different metabolic pathways for VFA productions in both process schemes.

## Figures and Tables

**Table 1:** Comparison of VFA yield in this study to literature.

Substrate	Hydrothermal Process Condition	Hydrothermal Process Severity Index	Fermentation Condition	VFA Yield	Increase in VFA	Reference
Food waste from canteen	160 °C, 30 min	3.2	Batch, 30 °C, 15 days	910 mg/g VS_removed_	55%	[10]
Restaurant, canteen, dining hall food waste	160 °C, 20 min	3.1	Batch, Fermentation: 35 °C, 48 h	450 mg/g VS	240%	[12]
Food waste from canteen	160 °C, 30 min	3.2	Batch, 30 °C, 21 days	740 mg/g VSS_removed_	130%	[11]
Kitchen waste from canteen	120 °C, 50 min	2.3	Batch, 35 °C, 21 days	32 mg/g VS	No increase	[14]
Kitchen waste from canteen	160 °C, 120 min	3.8	-	280 mmol/L	120%	[21]
				1.6 mmol/gVSS		
SSO	170 °C, 30 min	3.5	Semi-continuous, fed once a day, 38 °C, HRT = 3 days	433 mg/gVSS	135%	This study
				570 mg/gVSS_removed_		

Note: Severity Index = log {exp[(T − 100)/14.75] × t }, where T is the pretreatment temperature (°C), and t is the holding time (min) [13].

**Table 2:** The characteristics of SSO and inoculum.

Parameters	SSO	Inoculum
pH	5.55 ± 0.1	7.9 ± 0.1
TS mg/L	65,300 ± 1900	15,700 ± 100
VS mg/L	47,900 ± 1304	9250 ± 150
VS/TS (%)	72 ± 0.3	59 ± 1
TSS mg/L	62,000 ± 3500	15,400 ± 300
VSS mg/L	45,400 ± 2000	10,000 ± 200
VSS/TSS (%)	72.9 ± 1.9	65.2 ± 0.3
TCOD mg/L	99,200 ± 3200	18,000 ± 200
SCOD mg/L	32,500 ± 900	500 ± 70
Total carbohydrates (mg/L glucose)	12,500 ± 450	NA
Soluble carbohydrates (mg/L glucose)	450 ± 30	NA
Total protein mg/L	1600 ± 150	NA
Soluble protein mg/L	100 ± 15	NA
Total nitrogen (mg/L N)	3100 ± 607	1300 ± 40
Total phosphate (mg/L PO_4_^−^)	1250 ± 354	1350 ± 30
Alkalinity (mg/L CaCO3)	5600 ± 300	4700 ± 15
Ammonia (NH_3_-N)	900 ± 300	700 ± 50
VFA (mg/L HoAc)	8400 ± 680	680 ± 20

Total solids (TS), volatile solids (VS), total suspended solids (TSS), volatile suspended solids (VSS), total chemical oxygen demand (TCOD), soluble chemical oxygen demand (SCOD), volatile fatty acids (VFA), source separated organics (SSO).

## Supplementary Materials

The following are available online. Table S1. Soluble chemical oxygen demand (SCOD) and volatile fatty acid (VFA) yields. Figure S1. (a) Suspended solid reduction efficiency and (b) degree of solubilization of COD, carbohydrate, and protein in system-1 and system-2. (TSS: Total Suspended Solids, VSS: Volatile Suspended Solids, COD: Chemical Oxygen Demand). Figure S2. Ammonia concentration of raw, treated, and fermented source separated organics (SSO) samples.
