# Supplementary material for: Comparison of Two Process Schemes Combining Hydrothermal Treatment and Acidogenic Fermentation of Source-Separated Organics
Source: Molecules. 2019 Apr 13;24(8):1466. doi: 10.3390/molecules24081466 (PMC6514947; doi:10.3390/molecules24081466)
Supplement: Supplementary file 1 [file molecules-24-01466-s001.pdf]

Table 1. Soluble chemical oxygen demand (SCOD) and volatile fatty acid (VFA) yields.

Sample		SCOD Yield (mg/g VSS)	Increase in SCOD Yield (%)	VFA Yield (mg/g VSS)	Increase in VFA Yield (%)
**System-1**	Fermentation	288 ± 13	40 ± 3	149 ± 7	81 ± 2
	Hydrothermal post-treatment	478 ± 19	67 ± 3	184 ± 10	100 ± 3
**System-2**	Hydrothermal pre-treatment	363 ± 30	51 ± 4	34 ± 4	18 ± 2%
	Fermentation	521 ± 36	73 ± 5	249 ± 14	135 ± 5

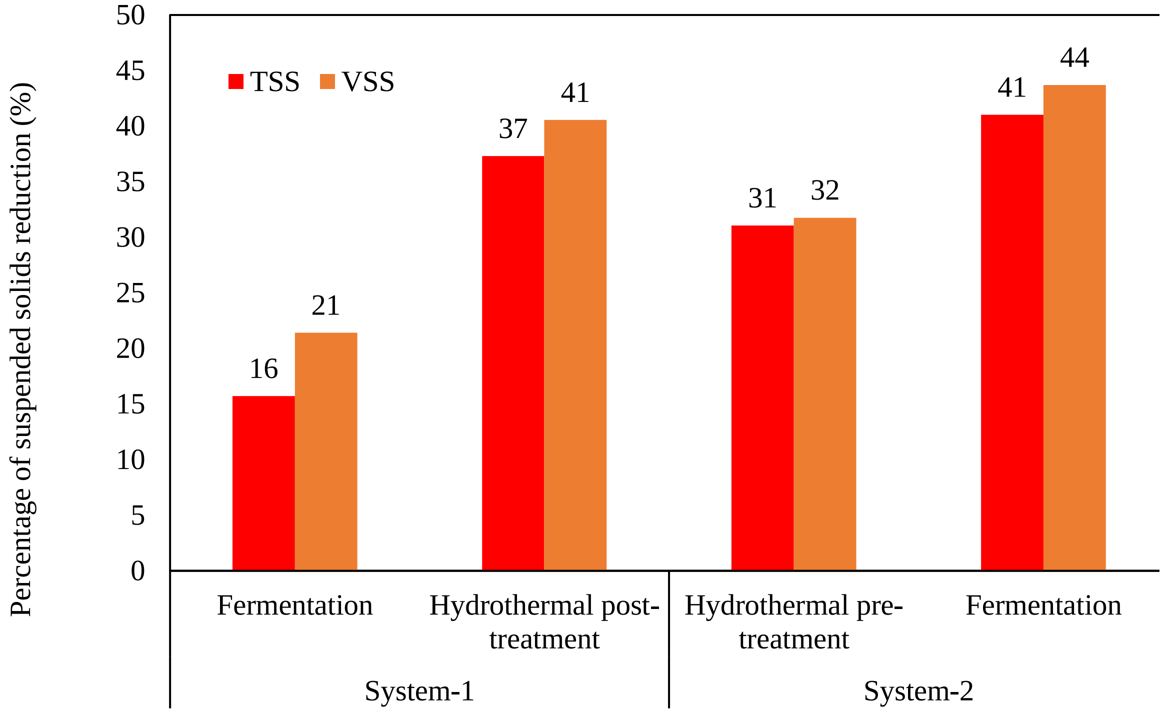

(**a**)

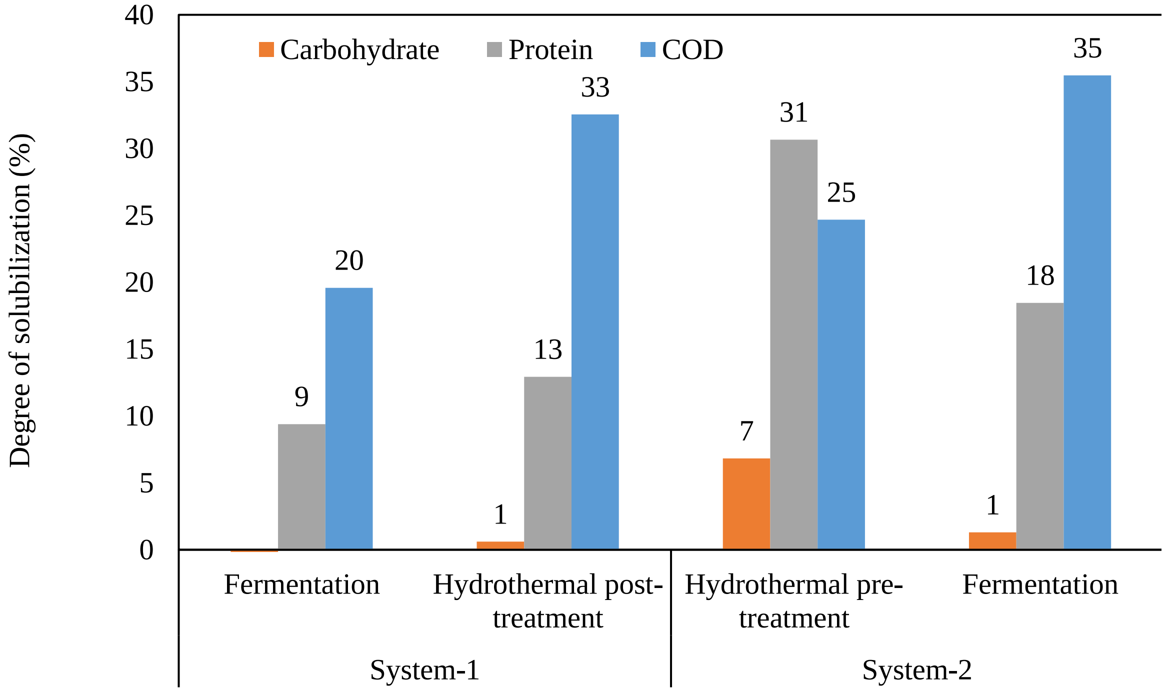

(**b**)

Figure 1. (**a**) Suspended solid reduction efficiency and (**b**) degree of solubilization of COD, carbohydrate, and protein in system-1 and system-2. (TSS: Total Suspended Solids, VSS: Volatile Suspended Solids, COD: Chemical Oxygen Demand)

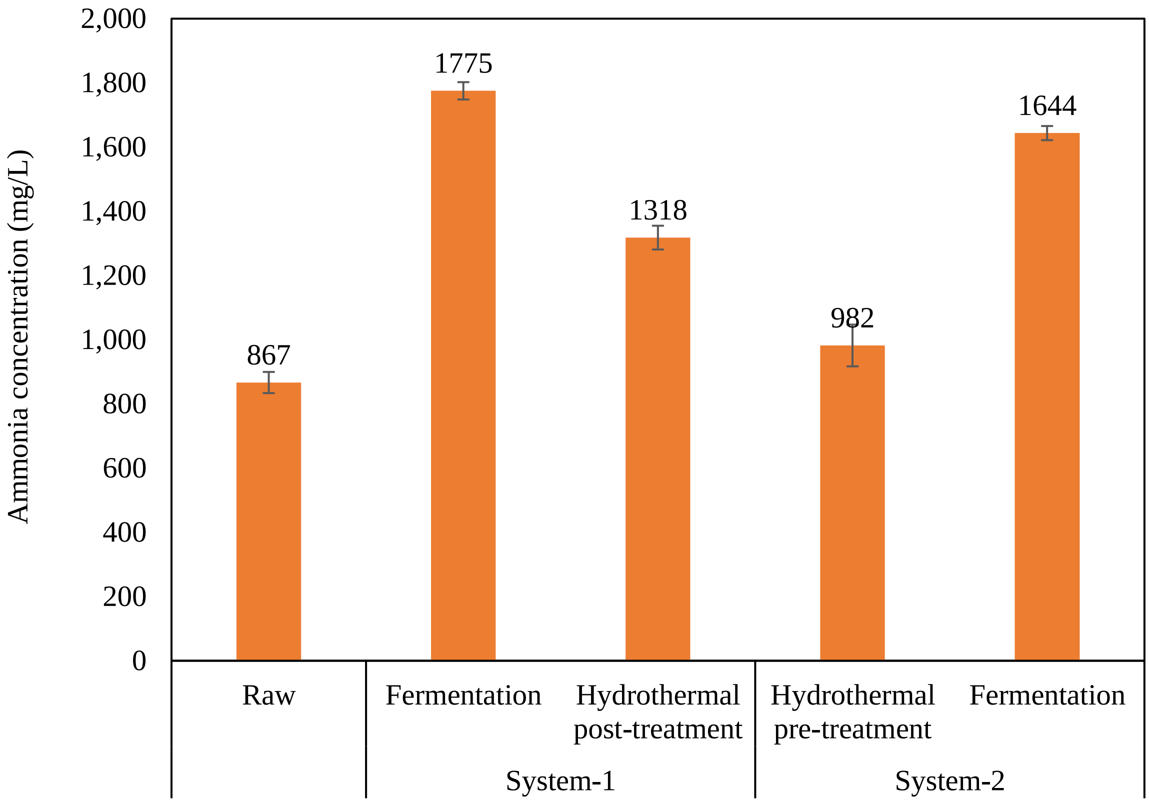

Figure 2. Ammonia concentration of raw, treated, and fermented source separated organics (SSO) samples.
